# In Vitro and Anti-Inflammatory Activity Evaluation Nanofibers from a Breath Mask and Filter Based on Polyurethane and Polyvinylidene Fluoride

**DOI:** 10.3390/polym15244650

**Published:** 2023-12-08

**Authors:** Kyu oh Kim

**Affiliations:** Department of Fiber System Engineering, Dankook University, 152, Jookjeon-ro, Suji-gu, Yongin-si 448-701, Gyeonggi-do, Republic of Korea; affablekim@gmail.com

**Keywords:** nanofiber (NF), cytotoxicity, polyurethane (PU), polyvinylidene fluoride (PVDF), DMSO stocks, nitric oxide production, protein expression

## Abstract

Nanofiber (NF) products exhibit outstanding performances in materials science, textiles, and medicine that cannot be realized using conventional technologies. However, the safety of such products is debated because of the potential health risks that nanomaterials pose and the lack of standardized guidelines for the safety evaluation of NF products. The global safety evaluations of nanomaterials have focused on evaluating the cytotoxicity of low-dimensional materials, including nanoparticles and nanotubes, based on OECD (Organization for Economic Co-operation and Development) criteria. NFs are one-dimensional materials with nanometer diameters and considerable lengths. Many fibers are applied in a densely woven web-like form, so assessing cellular penetration and fiber toxicity using the same methods is inappropriate. This study verifies the safety of the polyurethane (PU) and polyvinylidene fluoride (PVDF) polymers currently applied in filters and masks. To this end, polymer NFs were collected from each product, and the NFs were compared with reference samples using FT-IR and Raman spectroscopy. For the safety evaluation, DMSO stocks of varying concentrations of PVDF and PU NFs (at 0.5, 1, 5, and 10 μg/mL) were prepared. The cytotoxicity and inhibitory effects on nitric oxide production and protein expression obtained via Western blot were identified.

## 1. Introduction

Nanoparticles have diameters ranging from 1–100 nm, which are larger than atoms and smaller than cells. They are thus very small objects, approximately 1/100,000 the size of human hair [1,2]. Thus, an identical material in nano size acquires unique properties such as increased strength or electrical conductivity. Nanomaterials can readily alter the electrochemical properties on their surfaces, and using this, a diversity of nanomaterials can be fabricated according to their intended use [3,4]. Thus, they have been applied in various fields, from chemical materials to automobiles, information and communications technology, environmental energy, and biomedicine. Although nanomaterials are useful in daily life because of their unique properties, they have given rise to nano hazards. Owing to their small size, nanomaterials can more easily penetrate the human body and induce toxicity [5,6,7,8,9,10]. Safety assessments are critical because the cytotoxic potential of nanomaterials may vary according to various changes in physicochemical properties. Nanofibers (NFs) are defined as a type of nanomaterial comprising organic polymers in a 2D membrane form [11].

The most representative method for fabricating nanoscale NFs is electrospinning, a technique developed in the early 20th century that has been most notably applied in the filtration industry [12]. Electrospinning is a simple and rapid, high-performance technique for fabricating NFs using polymers. The technique applies a strong electromagnetic force at ~20–30 kV to ionize a polymer solution. The device has a polymer solution on one side and its opposite charge on the other, so a narrow jet of polymer solution called a Taylor cone is formed as polymer solutions tend to migrate towards opposite charges, forming 1D fibers with nanoscale diameters. The resultant formation of a membrane with a large surface area and porosity can be applied to high-performance filters, sensor components, biomaterials, and electrode separators [13,14,15,16,17,18,19,20,21,22,23,24]. However, a standard method for verifying the effects of the currently manufactured and commercialized NF products on the human body and determining potential hazards is lacking, and only a few studies have investigated the host response at the interface generated when NFs enter and accumulate in the human body. This has created the need for research on evaluating the safety of NF products. 

This study conducted a cytotoxicity assay using a cell line for validation before clinical trials to determine the effects of wearing a mask containing NFs on the human respiratory system [25,26]. A cytotoxicity assay is a biological assessment determining whether compounds are nontoxic and biocompatible. An in vitro analysis precedes an in vivo analysis as it gives rapid results and high reproducibility, and in vitro assays are widely used as a pre-validation test with the advantages of a reduced number of animals in subsequent animal studies and increased clinical accessibility, such as defining the concentrations of compounds. Cellular growth, regeneration, and morphological changes in cells are examined and evaluated in cytotoxicity assays. While there are several evaluation methods, the MTT assay, with its wide spectrum of use, has been applied for testing the cytotoxicity in this study.

Inflammation is a natural biological response to human tissue damage induced by various stimuli, from infection to chemical substances and immune responses. Macrophages play central roles in inflammatory responses, and through their phagocytosis, cytotoxicity, and cell-killing ability, they play a key role in the defense mechanism of the host. Lipopolysaccharides (LPSs) are generated in the cell walls of Gram-negative bacteria and have a powerful ability to induce inflammatory cytokines [27,28]. The LPS activity increases the production of inflammatory mediators such as nitric oxide (NO), tumor necrosis factor-α (TNF-α), interleukin (IL), prostaglandin (PG), and leukotriene. LPS-activated RAW 264.7 macrophages are widely used in exploring anti-inflammatory materials. Upon LPS stimulation, RAW 264.7 cells increase the secretion of cytokines such as IL-1β, IL-6, TNF-α, and NO. The production of NO employs nitric oxide synthase (NOS) and L-arginine substrates. The important roles of NOS include the control of vascular tone, neurotransmission, microbial removal, and homeostatic mechanisms. Inducible NOS (iNOS) is expressed upon stress or via inflammatory cytokines such as IL-1β, IL-6, and TNF-α; these cytokines activate macrophages and act as mediators for inducing acute and chronic inflammatory responses. Therefore, controlling such inflammatory cytokines and various mediators is critical for developing drugs for various inflammatory diseases, although they may also be key targets in creating anti-inflammatory materials.

This study verified the safety of the commercialized PVDF and PU NFs in currently available filters and masks. To this end, NFs were collected from sample products, and their physical properties were evaluated using Fourier-transform infrared (FT-IR) and Raman spectroscopy. Cytotoxicity assay and an anti-inflammatory investigation were conducted for a safety evaluation. The results confirm the anti-inflammatory effects of NF products and present the applicability of NFs as functional materials. These results provide basic data that can be applied to several different fields.

## 2. Materials and Methods

### 2.1. Materials

Electrospun polyvinylidene difluoride (PVDF or PVDF NFs) for a breath mask and PU (or PU NFs) for the breathable waterproof membrane were purchased from Lemon Corporation (Gumi, Republic of Korea). The nanofibers contained in the product were separated and used. The physical properties are summarized in Table 1. All samples were air-dried before characterization.

### 2.2. Fourier-Transform Infrared Spectroscopy

FT-IR spectra analysis was conducted using a Nicolet iN10MX (ThermoScientific, Waltham, MA, USA) to confirm the structural analysis of the PVDF NFs and PU NFs. The FT-IR spectra were obtained from KBr pellets, which were prepared by mixing KBr powder and the sample at a ratio of about 100:1 and grinding in an agate mortar to form a well-dispersed mixture, then pressing into a pellet with a pellet molding apparatus. The sample was analyzed over a 4000–715 cm^−1^ range (collection mode: Transmission, collection time: 0.113 s, Detector: array, up to 10 steps/second at 16 cm resolution equivalent to 1.2 × 1.2 mm in 4.5 min) to confirm the presence of the characteristic peaks of the PVDF and PU NFs and the changes in peak positions and shapes.

### 2.3. Raman Spectroscopy

Raman spectroscopic measurements were carried out using a Raman microspectrometer xplora plus (HORIBA Scientific Corp., Kyoto, Japan) equipped with an air-cooled Cobolt 08 DPL laser source (532 nm) with an output power of 105 mW and a Ge detector cooled by liquid nitrogen. The detector spectra range was 1050~3150 cm^−1^, monitored at the base of a quartz plate supporting PVDF and PU NFs, and was used for Raman measurement. 

### 2.4. Cell Viability

The commercially available research materials necessary for the study include reagents such as 3-(4,5-dimethythiazol-2-yl)-2,5-diphenyl tetrazolium bromide (MTT), dimethyl sulfoxide (DMSO), LPS, recombinant human TNF-α (rhTNF-α) purchased from Sigma Aldrich.co (St. Louis, MO, USA) and an ELISA reader (BioTek, Winooski, VT, USA). 

PU and PVDF NFs were tested in several solvents and found to dissolve most efficiently in DMSO. Therefore, DMSO stocks with varying concentrations of PU and PVDF NFs (0.5, 1, 5, and 10 μg/mL) were prepared, and an equal amount of each stock was used in the cellular assay. 

The human immortalized keratinocyte cell line (HaCaT) is widely used in skin physiology and differentiation studies. HaCaT cells were obtained from a government research center, and the experiment was conducted with relatively short passages (the cells were passaged no more than 10 times during the course of the experiment [33]. The RAW 264.7 (RAW) cell line is derived from BALB/c mice infected with the Abelson leukemia virus, which is popular as a monocyte/macrophage model [34]. RAW cells were obtained from the Korean Cell Line Bank (KCLB), and cells with short (~10) passages were applied in the experiment. The two types of cells were cultured in DMEM (HyClone, PA, USA) containing 10% heat-inactivated FBS (HyClone, PA, USA) and 100 U/mL penicillin/streptomycin (HyClone, PA, USA) in a 37 °C, 5% CO_2_ incubator. The cells were passaged every two to three days.

An MTT assay was conducted to measure cell viability. In an MTT assay, which relies on the ability of the dehydrogenase in cellular mitochondria to convert tetrazolium, a soluble, yellow substrate, into insoluble, blue-violet formazan, the absorbance indicates the amount of reduced formazan in proportion to the number of cells in each well. Cells in the MTT assay were seeded at 1–2 × 10^5^ cells/mL by counting only the viable cells after staining with 0.4% trypan blue. A hemacytometer was used for cell counting. At confluency <~80%, the cells were treated with NFs, LPS, or TNF-α. After 24-h culture, 40 μL of the culture solution was removed. Then, 40 μL of 5 mg/mL MTT reagent was added, and the cells were cultured for 3 h [35]. After completely removing the supernatant, the cells were dissolved in 40 μL of DMSO on a shaker for ~20 min. The absorbance was measured at 590 nm using an ELISA reader. Triplicate measurements were conducted for all test groups, and statistical significance was tested using the Student’s *t*-test with the significance level set at *p* < 0.05 [36].

### 2.5. Inhibitory Effects on Nitric Oxide (NO) Production

The level of NO_2_ present in the culture solution was measured using the Griess reagent to measure the amount of NO produced by RAW 264.7 cells. After seeding RAW 264.7 cells in a 6-well plate at 5 × 10^5^ cells/well, the cells were cultured for 24 h. Next, the cells were washed twice with 1× PBS and treated with 1 μg/mL of LPS, apart from the control group. After 1 h, the cells were treated with varying concentrations of sample solutions, and the supernatant was collected after a further 24-h culture. An equal volume of the Griess reagent was added for a 10-min reaction in a 96-well plate, and the absorbance was measured at 540 nm. The inhibitory effects on NO production were expressed as the rate of reduction in absorbance for the sample and non-sample groups.

### 2.6. Western Blot

RAW 264.7 cells were seeded in a 6-well plate at 5 × 10^5^ cells/well and stabilized through 24 h culturing to examine the iNOS and COX-2 activities. After removing the medium, the cells were treated with 1 μg/mL of LPS and, subsequently, with culture medium with varying concentrations of NFs. After 24-h culture, the medium was removed, and the cells were washed twice with PBS. Then, the cells were dissolved in 100 μL of a 10 mL solution of radio-immunoprecipitation assay (RIPA) buffer with complete mini 1 tab, followed by 20 min centrifugation at 4 °C and 16,110× *g*. The resulting supernatant was quantified using a BCA protein assay kit, and 20 μL of the collected proteins were electrophoresed using 10% SDS-PAGE. The separated proteins were transferred to the PVDF NFs and left in a blocking buffer (5% skim milk in TBST) at 25 °C for 1 h. The diluted primary antibodies to iNOS, COX-2, and β-actin were applied at 4 °C overnight, followed by three times washing with tris-buffered saline and tween 20 (TBST) in 10-min intervals. The secondary antibodies, anti-rabbit to iNOS and COX-2 and anti-mouse for β-actin, were diluted at 1:1000 for a 2-h reaction at room temperature. The bands were identified and quantified using an LAS 4000 device after washing with TBST three times.

## 3. Results and Discussion

### 3.1. Spectroscopic Investigation of Electrospun PVDF and PU Nanofibers

**Figure 1:**
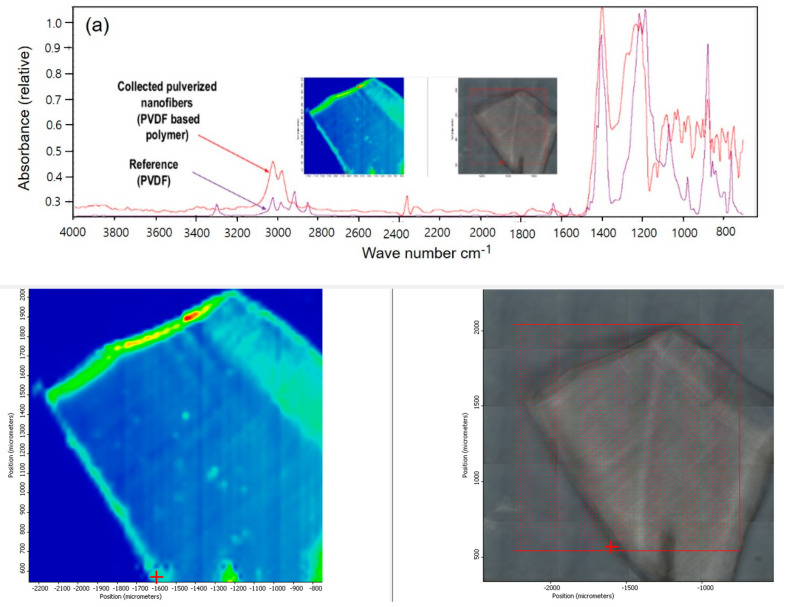

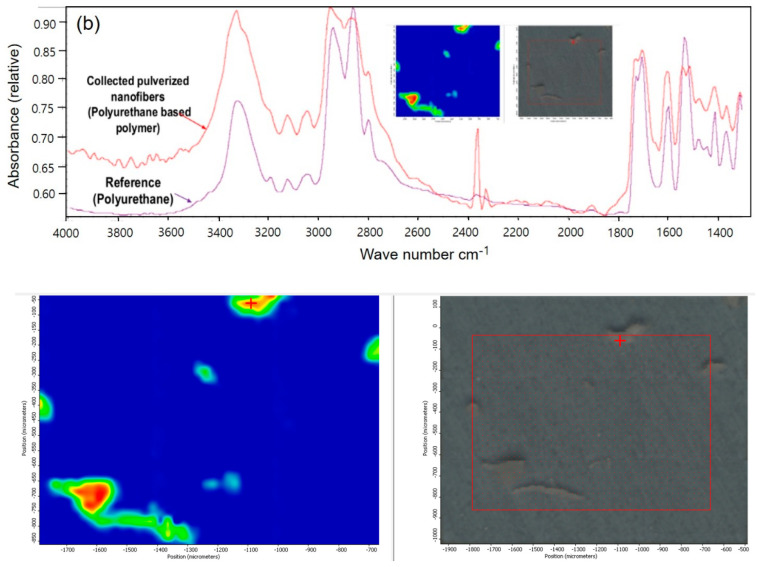
FT-IR spectra for (**a**) PVDF, (**b**) PU reference and nanofibers.

Table 1 lists the physical properties of the two different NFs used in this study. PVDF and PU NFs were analyzed using FT-IR and Raman spectroscopy to suggest two methods for evaluating the level of NFs dissociating from an NF product and entering the human body through the respiratory tract or other routes. Every molecule vibrates at a unique frequency. Applying light at a frequency equivalent to the vibration frequency of the molecule increases the vibration to cause special phenomena, including absorption or significant scattering of light. In other words, the identity of the molecule can be deduced if such special phenomena are detected upon applying light at a specific frequency to a given molecule. Figure 1 shows the PVDF and PU NF particles after grinding and the comparison between each respective reference line.

For PVDF, the most representative peaks were as follows: the characteristic absorption band of PVDF α-phase at 1072 cm^−1^ and β-phase at 1403 cm^−1^ (Figure 1a). The bands at 552 and 840 cm^−1^ are assigned to CF_2_ bending, and those at 796 cm^−1^ are assigned to the CF_2_ skeletal vibrational mode. The bands at 796 and 839 cm^−1^ are attributed to CH_2_ rocking. Splitting the band around 1180 cm^−1^ results in stretching vibrations of CF [37,38,39]. Figure 1b shows the presence of the NH, C=O, C=C, and C-N peaks at 3290, 1704, 1521, and 1600 cm^–1^, respectively, for PU. The peak at 2261 and 2380 cm^−1^ attributes the unreacted isocyanate group O=C=N [40]. The peaks of NF particles exhibit similar vibration behaviors to the reference peaks, as confirmed. 

**Figure 2:**
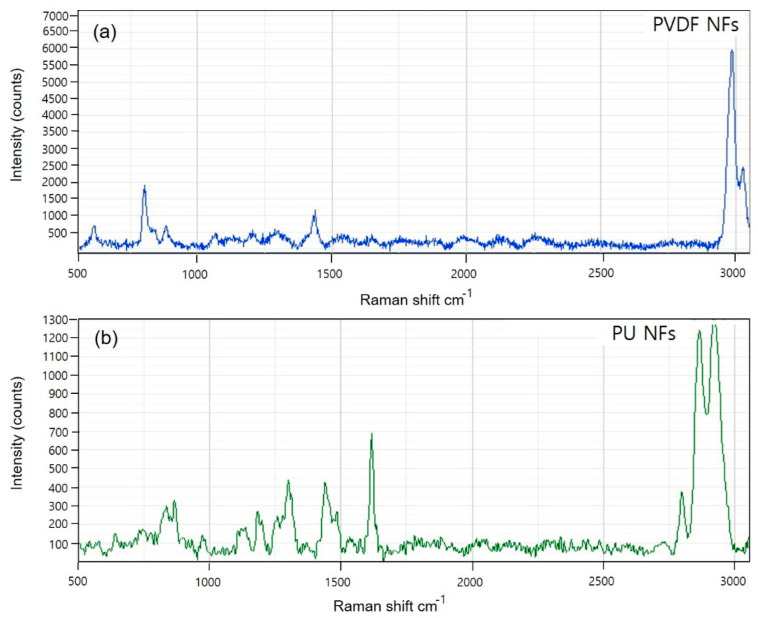
Raman spectra of PVDF (**a**) and PU (**b**) nanofibers on the breath mask and filter.

In addition, Raman spectrometry was used to evaluate the nanoparticles. Figure 2 shows the Raman spectra for PVDF and PU.

The PVDF spectra (Figure 2a) show that the crystal form is the α-phase, or form II, characterized by chain conformation. PVDF modes are observed at 480 and 611 cm^−1^. These modes are caused by CF_2_ vibrations. The modes at 513 cm^−1^ were attributed to the CF_2_ bending vibration, and the higher intensity band at 840 cm^−1^ was caused by an out-of-phase combination of the CH_2_ rocking and CF_2_ stretching modes [41]. These modes are β-phase or form I of PVDF, which are typical for the all-trans conformation of the PVDF chains. The all-trans conformations are attributed to the strain induced by swelling in the amorphous regions. The Raman spectrum of PU (Figure 2b) is characterized by three main urethane peaks: the C=O stretching vibration at 1620 cm^−1^ and the N single-bond H stretching and C single-bond H bending vibrations at 1450 and 1320 cm^−1^, respectively. The specific peaks of PVDF and PU NFs were identical to the reference peaks (Figure S1), and the potential use of the device in analyzing the dissociating nanoparticles was verified.

### 3.2. Anti-Inflammatory Activity Investigation of PVDF and PU NFs

**Figure 3:**
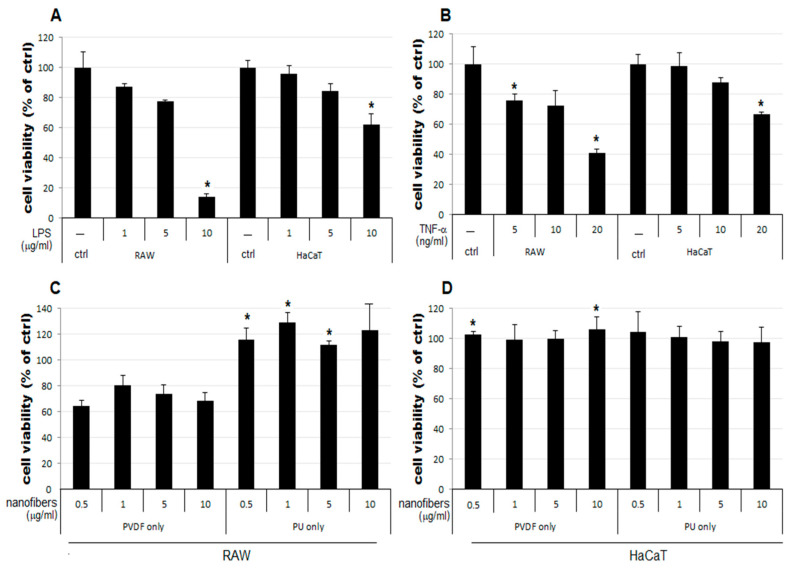
Results of cell viability with only PVDF NFs and PU NFs. Cell viability was decreased by LPS or TNF-α in a dose-dependent manner (**A**,**B**). Graphs show the results of the cell viability of nanofibers on RAW and HaCaT cells (**C**,**D**). The results are expressed as the mean ± SEM. * *p* < 0.05 vs. non-treated cells.

**Figure 4:**
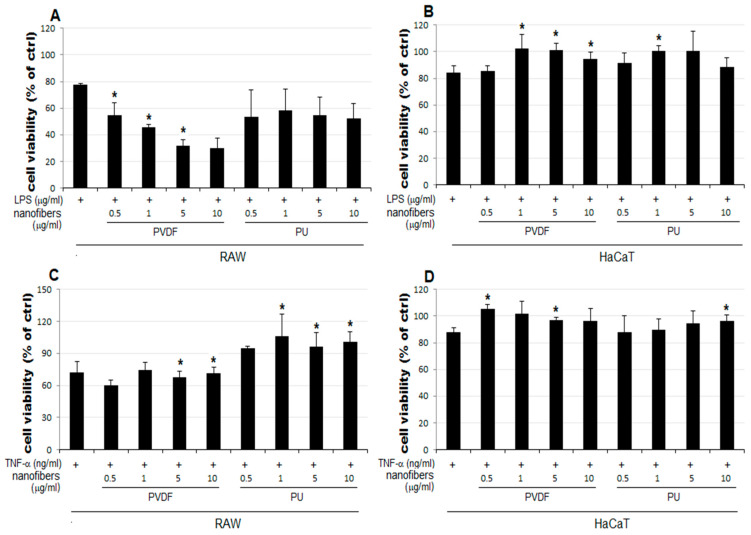
Results of the cell viability of NFs with LPS or TNF-α. HaCaT cells showed slightly higher cell viability with NFs than that with only stimuli (**B**,**D**). Cell viability is lowered with PVDF; however, in the case of PU, the cell viability was higher than the stimuli for only the treatment group (**A**,**C**). The results are expressed as the mean ± SEM. * *p* < 0.05 vs. cells treated with only LPS (**A**,**B**) or only TNF-α (**C**,**D**).

The result of the MTT assay demonstrated a concentration-dependent reduction in the LPS-treated groups of the two cell lines used as the positive control, compared to that of the control group (ctrl) (Figure 3A). The LPS 5 μg/mL group showed a reduction to 77% and 84% growth in RAW and HaCaT cells, respectively, compared to that of the control. Therefore, 5 μg/mL was set as the level of LPS stimulation in subsequent experiments to examine cell viability changes upon simultaneous treatment with NFs. In Figure 3C,D, RAW, and HaCaT cells were treated with PVDF and PU nanofibers to evaluate the inherent cytotoxicity of the nanofibers. No notable change in cellular growth is observed for HaCaT cells treated only with NFs (Figure 3D). In contrast, RAW cells treated only with PVDF NFs showed reduced cell viability. Those treated only with PU NFs showed significant overgrowth (Figure 3C). Groups treated with TNF-α as another positive control display a concentration-dependent reduction in cell viability (Figure 3B). As shown in Figure 3, the RAW cells treated only with PU NFs exhibit a higher level of cell viability compared to the control. It has been shown in the Rafal et al. study [42] that the cytotoxicity test we selected is more prominent than the MTT test. When evaluating PU nanofibers, due to the correlation between the tetrazolium salts used, high cell growth can be confirmed, unlike other biocompatible polymers. In contrast, cells treated simultaneously with LPS and PU NFs exhibit a trend toward a further decrease in cell viability compared to those treated only with LPS. A significant concentration-dependent reduction in cell viability is observed for cells treated with LPS and PVDF NFs (Figure 4A). The viability of RAW cells decreases significantly upon treatment with 5 and 10 μg/mL of PVDF NFs following the treatment with TNF-α; however, the difference in comparison with the cells treated with only PVDF NFs is not significant (Figure 4C). The treatment with 1, 5, and 10 μg/mL of PU NFs significantly increases the cell viability compared to the cells treated with TNF-α only (Figure 4C). For HaCaT cells, slightly increased or similar cell viability is observed across most concentrations of NFs compared to cells treated only with LPS or TNF-α (Figure 4B,D).

LPS is a component of the outer membrane of Gram-negative bacteria, and it is a well-known endotoxin that induces iNOS. This enzyme is responsible for producing various inflammatory cytokines and NO in macrophages, and it can increase the secretion of pro-inflammatory cytokines such as TNF-α, IL-1β, and IL-6 by stimulating macrophages or monocytes. Further, TNF-α is a cytokine produced in macrophages, lymphocytes, and leukocytes; it is not produced in a normal state, whereas its synthesis and secretion occur upon the stimulation of macrophages. The formation of NO plays a key role in killing bacteria or removing tumors; however, the inflammatory response, including edema and vascular permeability to NO produced by iNOS, has been shown to promote inflammation. The genetic expression of pro-inflammatory proteins such as TNF-α, IL-1β, and IL-6 is regulated via the activation of the nuclear factor kappa-light chain-enhancer of the activated B cells (NF-κB), which is a known transcription factor involved in inflammatory responses. NF-κB plays a crucial role in inflammatory responses by regulating the activation of iNOS. Assuming PU NFs play an inhibitory role in the synthesis of NO—a key mediator in LPS-mediated inflammation in RAW cells—it is necessary to determine changes in the amounts of cytokines that mediate inflammation (TNF-α, IL-1β, and IL-6) as inflammatory markers and verify changes in the amounts of iNOS and NF-κB proteins through Western blot analysis. 

In this study, changes in cell viability upon treatment with NFs were examined using RAW and HaCaT cells following the LPS- or TNF-α-induced inflammatory response. The results are summarized as follows:

1. The cell viability is not significantly affected in HaCaT cells treated only with NFs (Figure 4D). The cell viability increases in cells simultaneously treated with LPS or TNF-α and PU or PVDF NFs (Figure 4B,D).
2. The cell viability decreases to 64–80% in RAW cells treated only with PVDF NFs (Figure 4C). For RAW cells, the simultaneous treatment with LPS, TNF-α, and PVDF NFs led to a fall in cell viability. A significant reduction is observed upon simultaneous treatment with LPS and PVDF NFs in a concentration-dependent manner (Figure 4A).
3. The cell viability is at an excessive level in RAW cells treated with only PU NFs. In contrast, the simultaneous treatment with LPS and PU NFs does not increase cell viability (Figure 4A). Cells treated with TNF-α and PU NFs simultaneously show a significant increase in cell viability compared to those treated with only TNF-α (Figure 4B).

### 3.3. Inhibitory Effects of PVDF and PU NFs on Nitric Oxide Production

**Figure 5:**
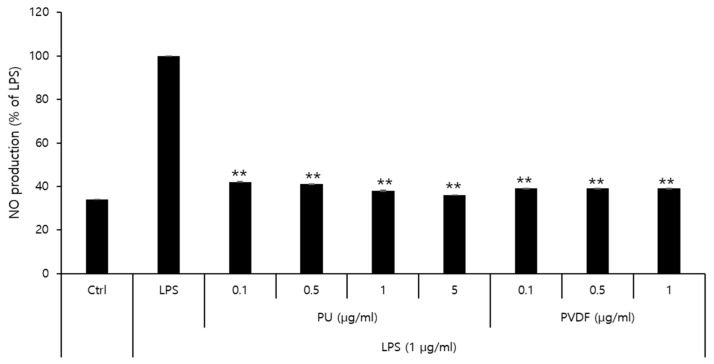
Effect of PU and PVDF NFs on NO production in LPS-induced RAW 264.7 cells. RAW 264.7 cells (5 × 10^5^ cells) were treated with PU and PVDF NFs and LPS (1 µg/mL) for 24 h. A statistically significant difference versus LPS was determined by ANOVA and Duncan’s multiple range test (Significant as compared to control; ** p < 0.01).

The effects of PU and PVDF NFs on NO production and their roles in inflammatory responses were determined. The MTT cytotoxicity assay demonstrated that the viability of RAW 264.7 cells decreased to 80% or lower after treatment with 5 μg/mL of PVDF NFs or 10 μg/mL of PU NFs. Thus, the final concentration of NO was set at 1 μg/mL and 5 μg/mL for PVDF and PU NFs, respectively. As shown in Figure 5, the level of NO expression in the LPS-treated group is higher compared to that of the control, whereas cells treated with PU or PVDF NFs exhibit reduced NO expression. The level of NO production was 38.0% at 5 μg/mL of PU NFs and 39.4% at 1 μg/mL of PVDF NFs, which indicated a 61.9% and 60.5% reduction, respectively. The inhibitory effects of PU and PVDF NFs on inflammatory responses were confirmed in RAW 264.7 cells. The safety of PU NFs was higher considering the high cell viability at low concentrations (0.1–1 μg/mL) of PVDF NFs in the previous cytotoxicity result.

### 3.4. Inhibitory Effects of PU NFs on iNOS and COX-2 Protein Expression

**Figure 6:**
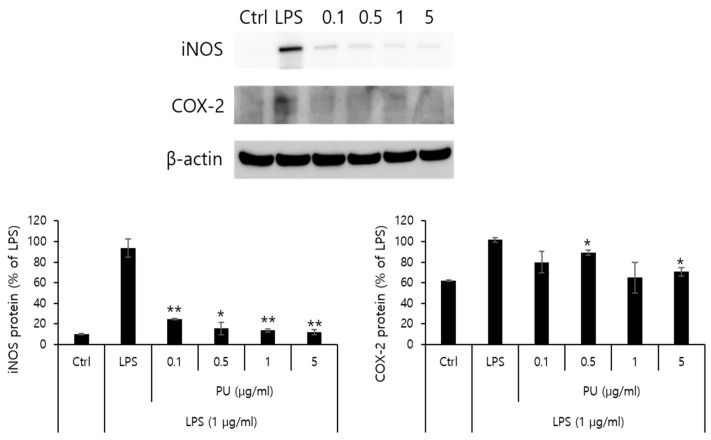
iNOS, COX-2 protein expression rate of PU NFs on macrophage cells (RAW 264.7). iNOS and COX-2 protein expression rates of PU NFs. After RAW 264.7 cells (5 × 10^5^ cells) were started in a serum-free medium for 1 h, the cells were treated with 0.1, 0.5, 1, and 5 µg/mL of PU NFs for 24 h. Statistically significant differences versus LPS by ANOVA and Duncan’s multiple range test. (Significant as compared to control. * p < 0.05, ** p < 0.01).

NO is an inflammatory marker synthesized by NOS using L-arginine. Three types of NOSs are present: endothelial NOS, neuronal NOS, and iNOS. The formation of NO by iNOS has an important pathological role. Another inflammatory marker, cyclooxygenase (COX), is an enzyme that converts arachidonic acid into prostaglandins. COX-1 and COX-2 have different patterns in different cells. Although COX-1 is involved in normal physiological functions in normal cells, COX-2 is expressed in areas of inflammatory responses. Further, PGE_2_, produced by COX-2, is an inflammatory mediator of pain and fever with a role in inflammatory and immune responses. This is closely related to angiogenesis. Western blot analysis was conducted to measure the inhibitory effects on protein expression of iNOS and COX-2 as inflammatory markers. The macrophage RAW 264.7 cells were treated with varying concentrations (0.1, 0.5, 1, and 5 μg/mL) of PU NFs. The inhibitory effects on the protein expression after 24 h are measured and shown in Figure 6. The positive control was β-actin from a housekeeping gene, whose expression rarely varies according to cell type or environment. Figure 6 shows that the protein expression of iNOS increased by LPS in RAW 264.7 cells decreased markedly to 24.6, 15.7, 13.6, and 12.1% after treatment with PU NFs at 0.1, 0.5, 1, and 5 μg/mL, respectively, in a concentration-dependent manner. At the same concentrations, the protein expression of COX-2 decreased to 79.8, 89.1, 64.9, and 70.6%, and the inhibition of protein expression was significant compared to the cells treated with LPS.

## 4. Conclusions

This study evaluated the safety of polyurethane (PU) and polyvinylidene fluoride (PVDF) nanofibers extracted from commercial filters and breathing masks. The chemical identities of the nanofibers were verified by comparing their FT-IR spectra with those of reference materials. Raman spectroscopy was used to investigate the properties of the nanofibers. The cytotoxicity assay and anti-inflammatory investigation were used for the safety evaluation. The nano hazards were evaluated in accordance with the verified anti-inflammatory effects using NF products. The results are listed below:

1. The cell viability is unaffected in HaCaT cells treated with only NFs (Figure 4D). The cell viability increased in cells simultaneously treated with LPS or TNF-α and PU or PVDF NFs.
2. The treatment with PU and PVDF NFs was effective in inhibiting the expression of inflammatory mediators in RAW 264.7 cells.
3. The LPS-induced iNOS expression in RAW 264.7 cells decreased after treatment with PU NFs at 0.1, 0.5, 1, and 5 μg/mL, indicating a high level of inhibition in a concentration-dependent manner. The inhibition of COX-2 expression was observed at equal concentrations, whereas the level of inhibition was higher compared to cells treated with LPS.

I believe that this study significantly contributes to the literature because it demonstrates the anti-inflammatory effects of NF products and the applicability of NFs as functional materials.

## Figures and Tables

**Table 1:** Physical properties of Electrospun PVDF and PU nanofibers.

Physical Property (Unit)	PVDF	PU	Test Method
Basic weight (g/m^2^)	4.1	3.8	ASTM D 3776 [29]
Air permeability (cm^3^/cm^2^/s)	1.02	1.2	ASTM D 737 [30]
Thickness (μm)	7.9	6	ASTM D 1777 [31]
Hydrostaucs (mmH_2_O)	700	5000	AATCC 127 [32]
Color	white	white	
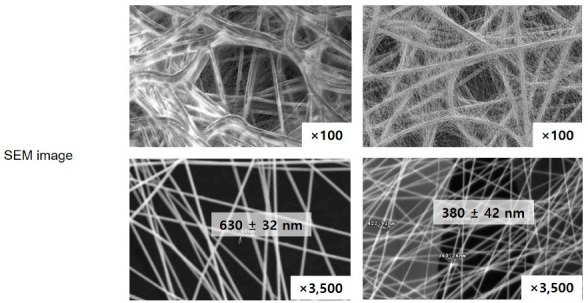			

## Data Availability

The data presented in this study are available on request from the corresponding author.

## Supplementary Materials

The following supporting information can be downloaded at https://www.mdpi.com/article/10.3390/polym15244650/s1, Figure S1: Raman spectra of PVDF and PU references.
