# Supplementary material for: In Vitro and Anti-Inflammatory Activity Evaluation Nanofibers from a Breath Mask and Filter Based on Polyurethane and Polyvinylidene Fluoride
Source: Polymers (Basel). 2023 Dec 8;15(24):4650. doi: 10.3390/polym15244650 (PMC10747216; doi:10.3390/polym15244650)
Supplement: Supplementary file 1 [file polymers-15-04650-s001.zip › polymers-2712466-supplementary.pdf]

Supplementary Materials

In Vitro and Anti-Inflammatory Activity Evaluation Nanofibers from a Breath Mask and Filter Based on Polyurethane and Polyvinylidene Fluoride

Kyu oh Kim

Department of Fiber System Engineering, Dankook University, 152, Jookjeon-ro, Suji-gu 448-701, Gyeonggi-do, Republic of Korea; affablekim@gmail.com

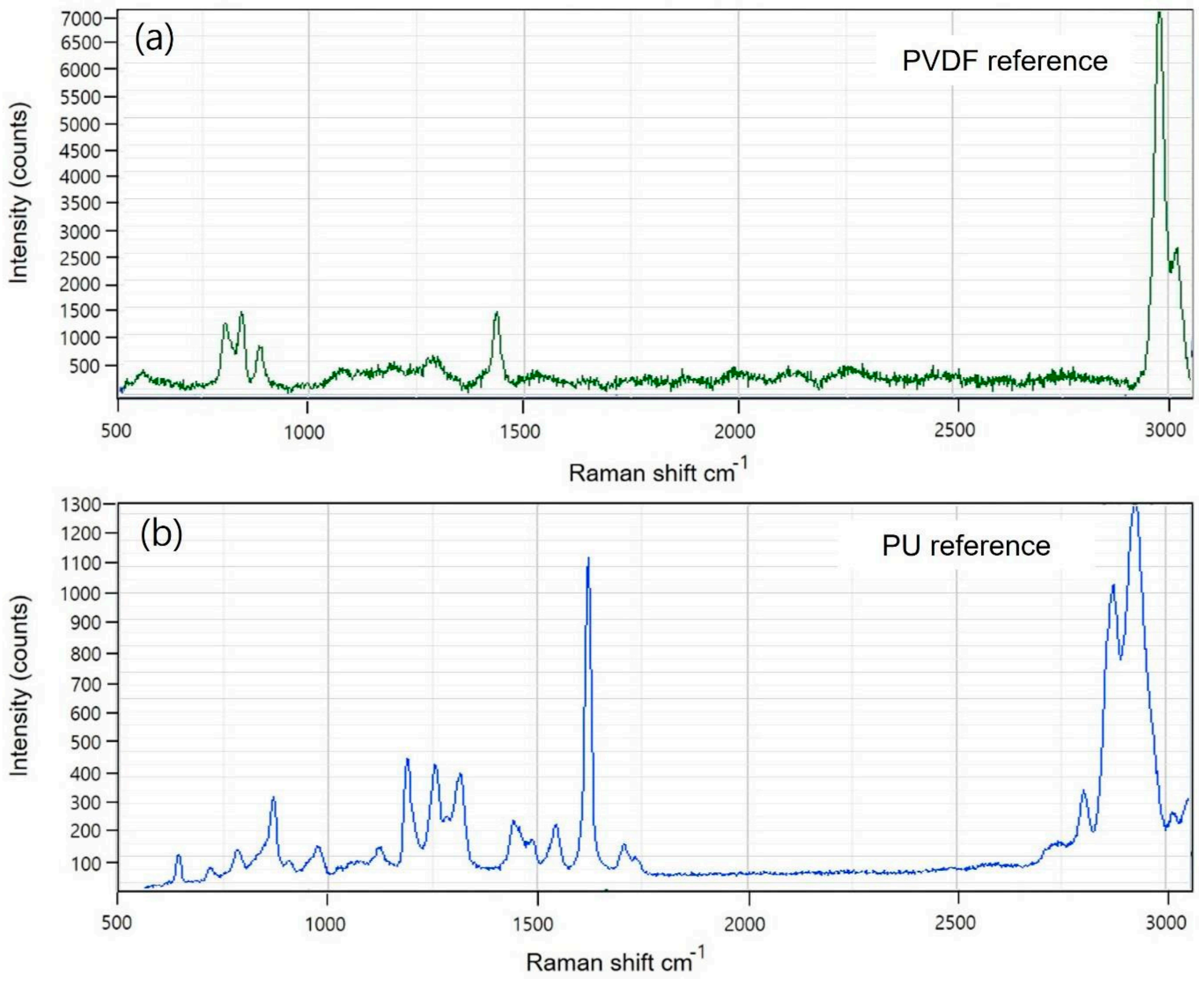

Figure S1. Raman spectra of PVDF and PU references.
